# Molecular characterization of the PhoPQ-PmrD-PmrAB mediated pathway regulating polymyxin B resistance in *Klebsiella pneumoniae *CG43

**DOI:** 10.1186/1423-0127-17-60

**Published:** 2010-07-24

**Authors:** Hsin-Yao Cheng, Yi-Fong Chen, Hwei-Ling Peng

**Affiliations:** 1 Department of Biological Science and Technology, National Chiao-Tung University, Hsin Chu, Taiwan, China; 2 Institute of Molecular Medicine and Bioengineering, National Chiao-Tung University, Hsin Chu, Taiwan, China

## Abstract

**Background:**

The cationic peptide antibiotic polymyxin has recently been reevaluated in the treatment of severe infections caused by gram negative bacteria.

**Methods:**

In this study, the genetic determinants for capsular polysaccharide level and lipopolysaccharide modification involved in polymyxin B resistance of the opportunistic pathogen *Klebsiella pneumoniae *were characterized. The expressional control of the genes responsible for the resistance was assessed by a LacZ reporter system. The PmrD connector-mediated regulation for the expression of *pmr *genes involved in polymyxin B resistance was also demonstrated by DNA EMSA, two-hybrid analysis and *in vitro *phosphor-transfer assay.

**Results:**

Deletion of the *rcsB*, which encoded an activator for the production of capsular polysaccharide, had a minor effect on *K. pneumoniae *resistance to polymyxin B. On the other hand, deletion of *ugd *or *pmrF *gene resulted in a drastic reduction of the resistance. The polymyxin B resistance was shown to be regulated by the two-component response regulators PhoP and PmrA at low magnesium and high iron, respectively. Similar to the control identified in *Salmonella*, expression of *pmrD *in *K. pneumoniae *was dependent on PhoP, the activated PmrD would then bind to PmrA to prolong the phosphorylation state of the PmrA, and eventually turn on the expression of *pmr *for the resistance to polymyxin B.

**Conclusions:**

The study reports a role of the capsular polysaccharide level and the *pmr *genes for *K. pneumoniae *resistance to polymyxin B. The PmrD connector-mediated pathway in governing the regulation of *pmr *expression was demonstrated. In comparison to the *pmr *regulation in *Salmonella*, PhoP in *K. pneumoniae *plays a major regulatory role in polymyxin B resistance.

## Background

*Klebsiella pneumoniae*, an important nosocomial pathogen, causes a wide range of infections including pneumonia, bacteremia, urinary tract infection, and sometimes even life-threatening septic shock [1]. The emergence of multi-drug resistant *K. pneumoniae *has reduced the efficacy of antibiotic treatments and prompted the reevaluation of previously but not currently applied antibiotics [2,3] or a combined therapy [4]. Polymyxins, originally isolated from *Bacillus polymyxa*, have emerged as promising candidates for the treatment of infections [5]. As a member of antimicrobial peptides (APs), the bactericidal agent exerts its effects by interacting with the lipopolysaccharide (LPS) of gram-negative bacteria. The polycationic peptide ring on polymyxin competes for and substitutes the calcium and magnesium bridges that stabilize LPS, thus disrupting the integrity of the outer membrane leading to cell death [5,6].

The *Klebsiella *capsular polysaccharide (CPS), which enabled the organism to escape from complement-mediated serum killing and phagocytosis [7,8], has been shown to physically hinder the binding of C3 complement [9] or polymyxin B [10]. The assembly and transport of *Klebsiella *CPS followed the *E. coli *Wzy-dependent pathway [11], in which mutations at *wza *encoding the translocon protein forming the complex responsible for CPS polymer translocation and export resulted in an inability to assemble a capsular layer on the cell surface [12]. The CPS biosynthesis in *K. pneumoniae *was transcriptionally regulated by the two-component system (2CS) RcsBCD [13] where the deletion of the response regulator encoding gene *rcsB *in *K. pneumoniae *caused a loss of mucoid phenotype and reduction in CPS production [14].

In *Escherichia coli *and *Salmonella enterica *serovar Typhimurium, polymyxin B resistance is achieved mainly through the expression of LPS modification enzymes, including PmrC, an aminotransferase for the decoration of the LPS with phosphoethanolamine [15] and the *pmrHFIJKLM *operon [16,17] (also called *pbgP *or *arn *operon [18,19]) encoding enzymes. Mutations at *pmrF*, which encoded a transferase for the addition of 4-aminoarabinose on bactoprenol phosphate, rendered *S. enterica *and *Yersinia pseudotuberculosis *more susceptible to polymyxin B [16,20]. The *S. enterica ugd *gene encodes an enzyme responsible for the supply of the amino sugar precursor _L_-aminoarabinose for LPS modifications and hence the Ugd activity is essential for the resistance to polymyxin B [21]. On the other hand, the *E. coli ugd *mutant with an impaired capsule also became highly susceptible to polymyxin B [22].

The 2CS PmrA/PmrB, consisting of the response regulator PmrA and its cognate sensor kinase PmrB, has been identified as a major regulatory system in polymyxin B resistance [23,24]. The resistance in *S. enterica *or *E. coli *has been shown to be inducible by the extracellular iron [25]. In addition to acidic pH [26], the role of ferric ions as a triggering signal for the expression of PmrA/PmrB has been demonstrated [23]. The 2CS PhoP/PhoQ which regulates the magnesium regulon [27] could also activate polymyxin B resistance under low magnesium in *S. enterica*, in which the PhoP/PhoPQ-dependent control is connected by the small basic protein PmrD. The expression of *pmrD *could be activated by PhoP while repressed by PmrA forming a feedback loop [28,29]. The activated PmrD could then bind to the phosphorylated PmrA leading to a persistent expression of the PmrA-activated genes [30].

The PmrD encoding gene was also identified in *E. coli *and *K. pneumoniae*. However, *pmrD *deletion in *E. coli *had no effect on the bacterial susceptibility to polymyxin B [25]. Recently, the PhoP-dependent expression of *pmrD *has also been demonstrated in *K. pneumoniae*. According to the predicted semi-conserved PhoP box in the *pmrD *upstream region, a direct binding of PhoP to the *pmrD *promoter for the regulation was speculated [31].

In this study, specific deletions of genetic loci involved in CPS biosynthesis and LPS modifications were introduced into *K. pneumoniae *CG43, a highly virulent clinical isolate of K2 serotype [32]. Involvement of the genetic determinants in polymyxin B resistance was investigated.

## Methods

### Plasmids, bacterial strains, and growth conditions

Bacterial strains and plasmids used in this study are listed in Table 1, and the primers used are listed in Table 2. *E. coli*, *K. pneumoniae *CG43 [32,33] and its derivatives were propagated at 37°C in Luria-Bertani (LB) broth or M9 minimal medium. Bacterial growth was assessed by OD_600_. The antibiotics used include ampicillin (100 μg/ml), chloramphenicol (35 μg/ml), kanamycin (25 μg/ml), tetracycline (12.5 μg/ml) and streptomycin (500 μg/ml). Polymyxin B sulfate salt (Sigma-Aldrich) was prepared as 1 unit/μl stock solution in PBS and stored at 4°C before use.

**Table 1:** Bacterial strains and plasmids used in this study

Strain or plasmid	Description	Reference or source
Strains		
*K. pneumponiae *		
CG43S3	CG43 Sm^r^	[14]
Δ*pmrF*	CG43S3Δ*pmrF *Sm^r^	This study
Δ*phoP*	CG43S3Δ*phoP *Sm^r^	This study
Δ*pmrD*	CG43S3Δ*pmrD *Sm^r^	This study
Δ*pmrA*	CG43S3Δ*pmrA *Sm^r^	This study
Δ*ugd*	CG43S3Δ*ugd *Sm^r^	This study
Δ*wza*	CG43S3Δ*wza *Sm^r^	This study
Δ*lacZ*	CG43S3Δ*lacZ *Sm^r^	[35]
Δ*lacZ*Δ*phoP*	CG43S3Δ*lacZ*Δ*phoP *Sm^r^	This study
Δ*lacZ*Δ*pmrD*	CG43S3Δ*lacZ*Δ*pmrD *Sm^r^	This study
Δ*lacZ*Δ*pmrA*	CG43S3Δ*lacZ*Δ*pmrA *Sm^r^	This study
Δ*pmrA*Δ*phoP*	CG43S3Δ*pmrA*Δ*phoP *Sm^r^	This study
Δ*rcsB *(B2202)	CG43S3Δ*rcsB *Sm^r^	[14]
Δ*pmrA*Δ*rcsB*	CG43S3Δ*pmrA*Δ*rcsB *Sm^r^	This study
Δ*pmrD*Δ*rcsB*	CG43S3Δ*pmrD*Δ*rcsB *Sm^r^	This study
Δ*phoP*Δ*rcsB*	CG43S3Δ*phoP*Δ*rcsB *Sm^r^	This study
		
*E. coli*		
S17-1λ*pir*	*hsdR recA pro *RP4-2 (Tc::Mu; Km::Tn*7*)(λ*pir*)	[34]
XL1-Blue MRF' Kan	Δ(*mcrA*)*183 *Δ(*mcrCB-hsdSMR-mrr*)*173 endA1 supE44 thi-1 recA1 gyrA96 relA1 lac *[F' *proAB lacI*^q^Z Δ*M15 *Tn*5 *(Kan^r^)]	Stratagene
BL21(DE3)	F^- ^*ompT hsdS_B_(r_B_^-^m_B_^-^) gal dcm trxB*15::*kan *(DE3)	Novagen
		
Plasmids		
yT&A	T/A-type PCR cloning vector, Ap^r^	Yeastern
pET30b	His-tagged protein expression vector, Km^r^	Novagen
pBT	Bait plasmid, *p15A *origin of replication, *lac-UV5 *promoter, λ-cI open reading frame, Cm^r^	Stratagene
pTRG	Target plasmid, *ColE1 *origin of replication, *lac-UV5 *promoter, *RNAP*αopen reading frame, Tc^r^,	Stratagene
pBT-LGF2	Control plasmid containing a fragment encoding the yeast transcriptional activator Gal4 fused with λ-cI, Cm^r^	Stratagene
pTRG-GAL11^P^	Control plasmid containing a fragment encoding a mutant form of Gal11 protein, called Gal11P, fused with RNAPα, Tc^r^	Stratagene
pKAS46	Suicide vector, *rpsL*, Ap^r^, Km^r^	[34]
pRK415	Shuttle vector, *mob*^+^, Tc^r^	[36]
placZ15	promoter selection vector, *lacZ*^+^, Cm^r^	[35]
pRK415-PmrF	1.3-kb fragment containing a *pmrF *allele cloned into pRK415, Tc^r^	This study
pRK415-RcsB	1.2-kb fragment containing the entire rcsB locus cloned into pRK415, Tc^r^	[39]
pRK415-PmrA	1.1-kb fragment containing a *pmrA *allele cloned into pRK415, Tc^r^	This study
pRK415-PhoP	900-bp fragment containing a *phoP *allele cloned into pRK415, Tc^r^	This study
pRK415-PmrD	550-bp fragment containing a *pmrD *allele cloned into pRK415, Tc^r^	This study
placZ15-PpmrH	500-bp fragment containing the upstream region of the *K. pneumoniae pbgP *genes cloned into placZ15, Cm^r^	This study
placZ15-PpmrD	350-bp fragment containing the upstream region of the *K. pneumoniae pmrD *genes cloned into placZ15, Cm^r^	This study
pET30b-PhoP	711-bp fragment encoding full-length PhoP cloned into pET30b, Km^r^	This study
pET30b-PhoPN	447-bp fragment encoding residues 1-149 of PhoP cloned into pET30b, Km^r^	This study
pET30b-PmrBC	828-bp fragment encoding residues 90-365 of PmrB cloned into pET30b, Km^r^	This study
pET-PmrA	669-bp fragment encoding full-length PmrA cloned into pET29b, Km^r^	This study
pET-PmrD	243-bp fragment encoding full-length PmrD cloned into pET29b, Km^r^	This study
pBT-PmrA	669-bp fragment encoding full-length RcsB cloned into pBT, Cm^r^	This study
pTRG-PmrD	243-bp fragment encoding full-length RcsA cloned into pTRG, Tc^r^	This study

**Table 2:** Oligonucleotide primers used in this study

Primer	**Sequence**^**a**^	Enzyme cleaved	Complementary position
ppmrF01	5'-GATGGAAAAGCTGAAGGCGATGG-3'	None	-161 relative to the *pmrF *start codon
ppmrF02	5'-CAGC**GATATC**ATACCCGGCGTC-3'	*Eco*RV	+1116 relative to the *pmrF *start codon
pmrA06	5'-GAG**CCATGG**TCTATTCCGTG-3'	*Nco*I	+682 relative to the *pmrA *start codon
pmrAp03	5'-CAATT**GGATCC**AGGGCTGTAC-3'	*Bam*HI	-424 relative to the *pmrA *start codon
phoP01	5'-CGCTCGCCGTTC**GGATCC**TG-3'	*Bam*HI	-171 relative to the *phoP *start codon
phoP02	5'-GCAAC**GGTACC**TTCATCAGCGC-3'	*Kpn*I	+729 relative to the *phoP *start codon
pmrDe02	5'-C**GAGCTC**GTGTTATTTGTCGGCGTTTGTC-3'	*Sac*I	+250 relative to the *pmrD *start codon
pmrDp01	5'-T**GGATCC**TTCATGACGCTCTCTC-3'	*Bam*HI	-278 relative to the *pmrD *start codon
pmrDp02	5'-CGCAC**AGATCT**GAAGCACGAC-3'	*Bgl*II	+75 relative to the *pmrD *start codon
pmrHp01	5'-TCT**GGATCC**TGGTCATTAATTGCCCGGC-3'	*Bam*HI	-425 relative to the *pmrH *start codon
pmrHp02	5'-CTT**AGATCT**CGCTCATCATCATCCTGTTC-3'	*Bgl*II	+34 relative to the *pmrH *start codon
phoP05	5'-GTAATGACAGCGGGAAGATATG-3'	None	+753 relative to the *phoP *start codon
phoP06	5'-CAGCCGTTTATATTTTGCGT-3'	None	-25 relative to the *phoP *start codon
pmrBe03	5'-T**GGATCC**TCGCAAGATCACCCGCC-3'	*Bam*HI	+283 relative to the *pmrB *start codon
pmrBe04	5'-C**AAGCTT**ATGGGTGCTGACGTTCTGAC-3'	*Hin*dIII	+1095 relative to the *pmrB *start codon
KP1760-1	5'-GGAATTC**CATATG**AAAATCTTAGTCATTGAA-3'	*Nde*I	+1 relative to the *pmrA *start codon
KP1760-2	5'-CCG**CTCGAG**CTATTCCGTGTCGATGTTGTT-3'	*Xho*I	+672 relative to the *pmrA *start codon
KP3573-1	5'-GGAATTC**CATATG**GAGTGGTGGGTAAAAAAA-3'	*Nde*I	+1 relative to the *pmrD *start codon
KP3573-2	5'-CCG**CTCGAG**TTTGTCGGCGTTTGTCCAACG-3'	*Xho*I	+243 relative to the *pmrD *start codon
pmrA10	5'-A**CTCGAG**CCATGGTCTATTCCGTG-3'	*Xho*I	+1 relative to the *pmrA *start codon
pmrA11	5'-AAT**GCGGCCGC**AATGAAAATCTTAGTC-3'	*Not*I	+672 relative to the *pmrA *start codon
pmrDe15	5'-AAA**GCGGCCGC**GATGGAGTGGTGGGTAAAAAAAGTA-3'	*Not*I	+1 relative to the *pmrD *start codon
pmrDe16	5'-TTT**CTCGAG**TGTGTTATTTGCCGGCGTTT-3'	*Xho*I	+243 relative to the *pmrD *start codon

^a ^The nucleotide sequence recognized by each restriction enzyme listed are in bold text.

### Construction of specific gene-deletion mutants

Specific gene deletion was individually introduced into the chromosome of *K. pneumoniae *CG43S3 by allelic exchange strategy [14]. In brief, two approximately 1000-bp DNA fragments flanking both sides of the deleted region were cloned into the suicide vector pKAS46 [34]. The resulting plasmid was then mobilized from *E. coli *S17-1 λ*pir *[34] to *K. pneumoniae *CG43S3, *K. pneumoniae *CG43S3Δ*lacZ *[35], or *K. pneumoniae *CG43S3Δ*rcsB *[14], by conjugation. The transconjugants were selected with ampicillin and kanamycin on M9 agar plates. Colonies were grown overnight in LB broth at 37°C and then spread onto an LB agar plate containing 500 μg/ml of streptomycin. The streptomycin-resistant and kanamycin-sensitive colonies were selected, and the deletion was verified by PCR and Southern analysis using gene-specific probe. The resulting *K. pneumoniae *mutants are listed Table 1.

To obtain the complementation plasmids, DNA fragments containing the coding sequence of *pmrA*, *phoP*, *pmrF*, or *pmrD *were PCR-amplified with primer sets pmrAp03/pmrA06, phoP01/phoP02, ppmrF01/ppmrF02 or pmrDp01/pmrDe02 (Table 2) and cloned into the shuttle vector pRK415 [36] to generate pRK415-PmrA, pRK415-PhoP, pRK415-PmrF and pRK415-PmrD (Table 1), respectively.

### Extraction and quantification of CPS

Bacterial CPS was extracted using the method described [37]. Briefly, 500 μl of overnight culture was mixed with 100 μl of 1% Zwittergent 3-14 (Sigma-Aldrich) in 100 mM citric acid (pH 2.0) and incubated at 50°C for 20 min. After centrifugation, 250 μl of the supernatant was used to precipitate CPS with 1 ml of absolute ethanol. The pellet was dissolved in 200 μl distilled water, and then 1,200 μl of 12.5 mM borax in H_2_SO_4 _was added. The mixture was vigorously mixed, boiled for 5 min, cooled, and then 20 μl 0.15% 3-hydroxydiphenol (Sigma-Aldrich) was added. OD_520 _was measured and the uronic acid content was determined from a standard curve of glucuronic acid and expressed as μg per 10^9 ^CFU.

### Polymyxin B resistance assay

**Figure 1:**
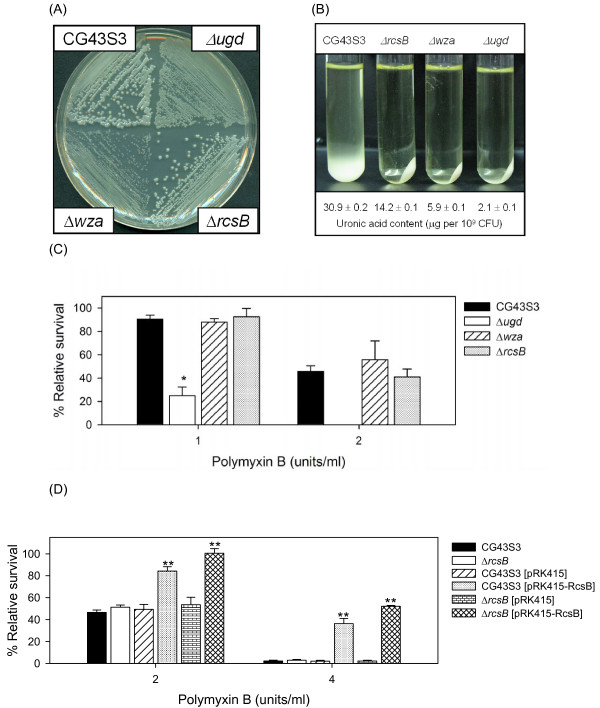
**Deletion effects of *ugd*, *wza *and *rcsB *genes on *Klebsiella *CPS production and resistance to polymyxin B**. **(A) **Comparison of colony morphology. The *K. pneumoniae *strains were streaked on an LB agar plate, incubated at 37°C overnight and photographed. **(B) **Sedimentation test. The strains were cultured overnight in LB broth at 37°C and subjected to centrifugation at 4,000 ×*g *for 5 min. Quantification of K2 CPS amounts of each strain is shown below the figure. Values are shown as averages ± standard deviations from triplicate samples. **(C) **Polymyxin resistance assay. The log-phased cultures of *K. pneumoniae *CG43S3, Δ*ugd*, Δ*wza *or Δ*rcsB *mutants were challenged with 1 or 2 units/ml of polymyxin B. **(D) **Polymyxin resistance assay. The log-phased culture of *K. pneumoniae *strains were challenged with 2 or 4 units/ml of polymyxin B. The survival rates are shown as the average ± standard deviations from triplicate samples. *, *P *< 0.01 compared to the parental strain CG43S3. **, *P *< 0.01 compared to each strain carrying pRK415.

**Figure 2:**
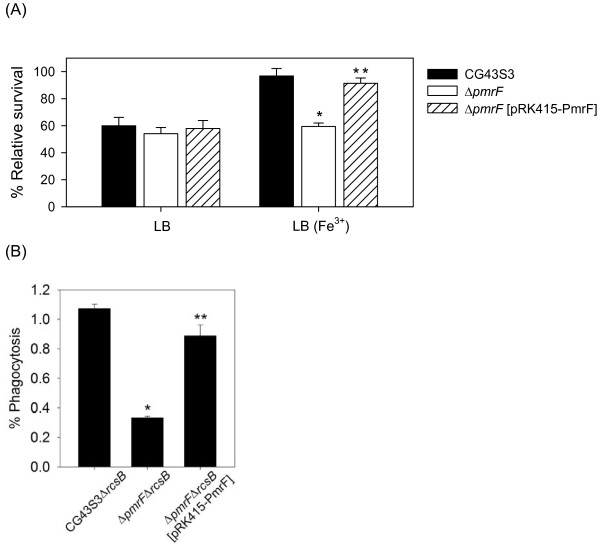
**Involvement of *K. pneumoniae pmrF *gene in polymyxin B resistance and intramacrophage survival**. **(A) **The log-phased cultures of *K. pneumoniae *CG43S3, the Δ*pmrF *mutant or Δ*pmrF *carrying pRK415-PmrF were grown in LB or LB supplemented with 1 mM Fe^3+ ^and then challenged with 2 units/ml of polymyxin B. The survival rates are shown as the average ± standard deviations from triplicate samples. **(B) **The survival rates of *K. pneumoniae *CG43S3Δ*rcsB*, the isogenic Δ*pmrF*Δ*rcsB *mutant, and Δ*pmrF*Δ*rcsB *mutant strain carrying the complementation plasmid pRK415-PmrF within the mouse macrophage RAW264.7 were determined. The results shown are relative survival rates which were calculated from the viable colony counts of intracellular bacteria divided by individual original inoculums. Values are shown as the average of five replicas. Error bars, standard deviations. *, *P *< 0.01 compared to each parental strain; **, *P *< 0.01 compared to each mutant strain carrying pRK415-PmrF.

**Figure 3:**
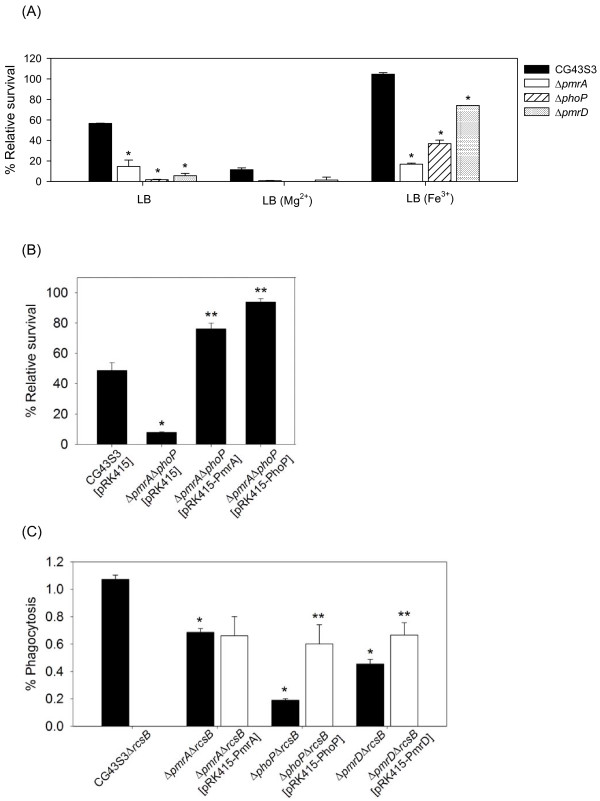
**Effects of *K. pneumoniae pmrA, pmrD *and *phoP *deletion and complementation in polymyxin B resistance and intramacrophage survival**. **(A) **The log-phased cultures of *K. pneumoniae *CG43S3, the Δ*pmrA*, Δ*pmrD *or Δ*phoP *mutants were grown in LB, LB supplemented with 10 mM Mg^2+ ^or LB supplemented with 1 mM Fe^3+ ^and then challenged with 2 units/ml of polymyxin B. The survival rates are shown as the average ± standard deviations from triplicate samples. **(B) **The log-phased cultures of *K. pneumoniae *CG43S3 carrying pRK415, the Δ*pmrA*Δ*phoP *mutant strains carrying pRK415, pRK415-PhoP or pRK415-PmrA were grown in LB and challenged with 2 units/ml of polymyxin B. The survival rates are shown as the average ± standard deviations from triplicate samples. **(C) **The survival rates of *K. pneumoniae *CG43S3Δ*rcsB*, the isogenic Δ*pmrA*Δ*rcsB*, Δ*phoP*Δ*rcsB *and Δ*pmrD*Δ*rcsB *mutants, and each mutant strain carrying the complementation plasmids pRK415-PmrA, pRK415-PhoP or pRK415-PmrD within the mouse macrophage RAW264.7 were determined. The results shown are relative survival rates which were calculated from the viable colony counts of intracellular bacteria divided by individual original inoculums. Values are shown as the average of five replicas. Error bars, standard deviations. *, *P *< 0.01 compared to each parental strain; **, *P *< 0.01 compared to each mutant strain carrying the complementation plasmid.

Polymyxin B resistance assay was performed essentially as described [10] with some modifications. In brief, the overnight-grown *K. pneumoniae *strains were washed twice with saline (0.85% NaCl solution, w/v) and subcultured in LB broth alone or supplemented with 1 mM FeCl_3 _or with 10 mM MgCl_2 _at 37°C. The log-phased (OD_600 _of 0.7) bacterial culture was then washed twice and a suspension containing ca. 2.5 × 10^4 ^CFU/ml in LB was prepared. Then, 100 μl of the suspension was placed in each well of a 96-well micro-titer plate and 100 μl PBS or PBS-diluted polymyxin B was added to each well to final concentrations of 0, 1, 2, or 4 units/ml of polymyxin B. The plate was incubated at 37°C for 1 h with shaking. Subsequently, 100 μl of the suspension was directly plated on LB agar plates and incubated at 37°C overnight to determine the number of viable bacteria. The survival rates were expressed as colony counts divided by the number of the same culture treated with PBS and multiplied by 100. The assays were performed thrice, and the results were shown as the average ± standard deviation from triplicate samples. The survival rates at 1 and 2 units/ml (Figure 1C) and at 2 units (Figure 2A and Figure 3AB) of polymyxin B were shown.

### Cell line, cell culture and phagocytosis assay

The mouse macrophage cell line RAW264.7 was cultivated in Dulbecco's Modified Eagle Medium (DMEM) (Gibco) supplemented with 10% fetal bovine serum (Gibco), 100 units/ml of penicillin and 100 μg/ml of streptomycin (Gibco) at 37°C under 5% CO_2_. The evaluation of bacterial phagocytosis was carried out as described with some modifications [9]. In brief, cells were washed, resuspended in DMEM containing 10% FBS, and approximately 10^6 ^cells per well were seeded in a 24 well tissue culture plate and incubated at 37°C for 16 h. Then 100 μl of the bacterial suspension (approximately 3 × 10^8 ^CFU/ml in PBS) was used to infect each well to obtain a ratio of ca. 30 bacteria per macrophage. After incubation for 2 h, the cells were washed thrice, then 1 ml of DMEM containing 100 μg/ml of gentamycin was added and incubated for another 2 h to kill the extracellular bacteria. Cells were washed thrice, 1 ml of 0.1% Triton X-100 was added and incubated at room temperature for 10 min with gentle shaking to disrupt the cell membrane. The cell lysate was diluted serially with PBS, plated onto LB agar plates and incubated overnight for determining viable bacteria count. The relative survival rates after phagocytosis were expressed as the colony counts of viable bacteria divided by those of the original inoculums and multiplied by 100. Three independent trials were performed, and the data shown were the average ± standard deviation from five replicas.

### Construction of reporter fusion plasmid and measurement of promoter activity

The approximately 350 or 500-bp DNA fragments containing the upstream region of the *K. pneumoniae pmrD *or *pmrHFIJKLM *gene cluster were PCR-amplified with primers pmrDp01/pmrDp02 or pmrHp01/pmrHp02 (Table 2), respectively and cloned in front of a promoter-less *lacZ *gene of the promoter selection plasmid placZ15 [35]. The resulting plasmids, placZ15-PpmrD and placZ15-PpmrH were mobilized from *E. coli *S17-1 λ*pir *to *K. pneumoniae *strains by conjugation. β-galactosidase activity was determined as previously described [35]. In brief, overnight cultures were washed twice with saline and subcultured in LB alone or supplemented with 10 mM MgCl_2_, 0.1 mM FeCl_3_, or 0.1 mM FeCl_3 _plus 0.3 mM ferric iron scavenger deferoxamine (Sigma-Aldrich) to mid-log phase (OD_600 _of 0.7). Then 100 μl of the culture was mixed with 900 μl of Z buffer (60 mM Na_2_HPO_4_, 40 mM NaH_2_PO_4_, 10 mM KCl, 1 mM MgSO_4_, 50 mM β-mercaptoethanol), 17 μl of 0.1% SDS, and 35 μl of chloroform and the mixture was shaken vigorously. After incubation at 30°C for 10 min, 200 μl of 4 mg/ml ONPG (o-nitrophenyl-β-D-galactopyranoside) (Sigma-Aldrich) was added. Upon the appearance of yellow color, the reaction was stopped by adding 500 μl 1 M Na_2_CO_3_. OD_420 _was recorded and the β-galactosidase activity was expressed as Miller units [38]. Each sample was assayed in triplicate, and at least three independent experiments were carried out. The data shown were calculated from one representative experiment and shown as the means and standard deviation from triplicate samples.

### Cloning, expression and purification of recombinant proteins

The DNA fragment of PhoP coding region was PCR amplified from the genomic DNA of *K. pneumoniae *CG43S3 with primers phoP05/phoP06 (Table 2). The amplified PCR products were cloned into the PCR cloning vector yT&A (Yeastern Biotech, Taiwan). The *Eco*RI/*Bam*HI and *Sal*I fragments from the resulting plasmid were then cloned individually into pET30b (Novagen, Madison, Wis) to generate pET30b-PhoP and pET30b-PhoPN to allow the in-frame fusion to the N-terminal His codons. Plasmid pET30b-PmrBC was constructed by cloning DNA fragments PCR-amplified with pmrBe03/pmrBe04 (Table 2) into a *Bam*HI/*Hin*dIII site on pET30b. Plasmids pET-PmrA and pET-PmrD (courtesy of Dr. Chinpan Chen, Academia Sinica, Taipei, Taiwan) were constructed by cloning DNA fragments PCR-amplified with KP1760-1/KP1760-2 and KP3573-1/KP3573-2 (Table 2) into an *Nde*I/*Xho*I site, respectively into pET29b. The resulting plasmids were transformed into *E. coli *BL21(DE3) (Invitrogen, USA), and the recombinant proteins were over-expressed by induction with 0.5 mM isopropyl 1-thio-β-D-galactopyranoside (IPTG) for 3 h at 37°C. The proteins were then purified from total cell lysate by affinity chromatography using His-Bind resin (Novagen, Madison, Wis). After purification, the eluent was dialyzed against 1× protein storage buffer (10 mM Tris-HCl pH 7.5, 138 mM NaCl, 2.7 mM KCl, and 10% glycerol) at 4°C overnight, followed by condensation with PEG20000, and the purity was determined by SDS-PAGE analysis.

### DNA electrophoretic mobility shift assay (EMSA)

EMSA was performed as previously described [14]. In brief, the DNA fragment encompassing the putative *pmrD *promoter region was obtained by PCR amplification and then end-labeled with [γ-^32^P]ATP by T4 polynucleotide kinase. The purified His-PhoP or His-PhoP_N149 _protein was mixed with the DNA probe in a 50-μl reaction mixture containing 20 mM Tris-HCl pH 8.0, 50 mM KCl, 1 mM MgCl_2_, 1 mM dithiothreitol, and 7.5 mM acetyl phosphate. The mixture was incubated at room temperature for 30 min, mixed with 0.1 volume of DNA loading dye, and then loaded onto a 5% nondenaturing polyacrylamide gel containing 5% glycerol in 0.5× TBE buffer (45 mM Tris-HCl pH 8.0, 45 mM boric acid, 1.0 mM EDTA). After electrophoresis at a constant current of 20 mA at 4°C, the result was detected by autoradiography.

### Bacterial two-hybrid assay

The bacterial two-hybrid assay was performed as described previously [20,30]. The DNA fragments encoding full-length PmrA and PmrD were PCR-amplified with primer pairs pmrA10/pmrA11 and pmrDe15/pmrDe16 (Table 2) respectively, and cloned into the 3' end of genes encoding the α subunit of RNA polymerase (RNAPα) domain on pBT and λ-cI repressor protein domain on pTRG. The resulting RNAPα-PmrA and λ-cI-PmrD encoding plasmids, pBT-PmrA and pTRG-PmrD, were confirmed by DNA sequencing. The positive control plasmids used were pTRG-Gal11^P ^and pBT-LGF2 (Stratagene). The pBT and pTRG derived plasmids were co-transformed into *E. coli *XL1-Blue MRF' Kan cells and selected on LB agar plates supplemented with 12.5 μg/ml tetracycline, 25 μg/ml chloramphenicol, and 50 μg/ml kanamycin. To investigate the protein-protein interaction *in vivo*, cells were grown until the OD_600 _reached 0.3 and then diluted serially (10^-1^, 10^-2^, 10^-3^, and 10^-4 ^order). Two-microliters of the bacterial culture were spotted onto LB agar plates supplemented with 350 μg/ml carbenicillin, 25 μg/ml chloramphenicol, 50 μg/ml kanamycin, 12.5 μg/ml tetracycline, 50 μg/ml X-gal (5-bromo-4-chloro-3-indolyl-β-D-galactopyranoside), and 20 μM IPTG. Growth of the bacterial cells was observed after incubation at 30°C for 36 h.

### *In vitro *phosphotransfer assay

The *in vitro *phosphotransfer assay was performed essentially as described [30]. The phospho-PmrB_C276 _protein was obtained by pre-incubation of His-PmrB_C276 _protein (5 μM) with 40 μCi of [γ-32P]ATP in 80 μl of 1× phosphorylation buffer (10 mM Tris-HCl, pH 7.5; 138 mM NaCl; 2.7 mM KCl; 1 mM MgCl_2_; 1 mM DTT) for 1 h at room temperature. The reaction mixture was then chilled on ice, and 5 μl of the mixture was removed and mixed with 2.5 μl of 5× SDS sample buffer as a reference sample. The phospho-PmrB_C276 _protein mixture (30 μl) was then mixed with equal volumes of 1× phosphorylation buffer containing either PmrA (10 μM) or PmrA with PmrD (each at 10 μM) to initiate the phosphotransfer reaction. A 10-μl aliquot was removed at specific time points, mixed with 2.5 μl of 5× SDS sample buffer to stop the reaction, and the samples were kept on ice until the performance of SDS-PAGE. After electrophoresis at 4°C, the signal was detected by autoradiography.

### Kinase/phosphatase and autokinase assay

The assays were performed essentially as described [30]. The recombinant protein His-PmrB_C276 _(2.5 μM) was incubated with His-PmrA (5 μM) alone or with His-PmrD (5 μM) for kinase/phosphatase assay or incubated with His-PmrD (5 μM) alone for autokinase assay. The reactions were carried out in 30 μl of 1× phosphorylation buffer with 3.75 μCi [γ-^32^P]ATP at room temperature and started with the addition of His-PmrB_C276_. An aliquot of 10-μl was removed at specific time points, mixed with 5× SDS sample buffer to stop the reaction, and the samples were kept on ice until the performance of SDS-PAGE. After electrophoresis at 4°C, the signal was detected by autoradiography.

### Statistical analysis

Student's *t *test was used to determine the significance of the differences between the CPS amounts and the levels of β-galactosidase activity. *P *values less than 0.01 were considered statistically significant.

## Results

### Reduced production of capsular polysaccharide had minor effect on polymyxin B resistance in *K. pneumoniae*

*K. pneumoniae *CG43 is a highly encapsulated virulent strain [32]. In order to verify the role of CPS in polymyxin B resistance, the Δ*ugd *and Δ*wza *mutants were generated by allelic exchange strategy, and their phenotype as well as the amount of CPS produced were compared with the parental strain CG43S3 and Δ*rcsB *mutant [14]. As shown in Figure 1A, the Δ*ugd *and Δ*wza *mutants formed apparently smaller colonies on LB agar plate compared with the glistering colony of the parental strain CG43S3. Although the colony morphology of the Δ*rcsB *mutant was indistinguishable from CG43S3, the CPS-deficient phenotype was evident as assessed using sedimentation assay and the amount of K2 CPS produced (Figure 1B). Deletion of *rcsB *resulted in an approximately 50% reduction of the CPS, while the Δ*wza *mutant produced less than 20% of that of its parental strain CG43S3. The CPS biosynthesis in Δ*ugd *mutant was almost abolished, indicating an indispensible role of Ugd in CPS biosynthesis. To investigate how the CPS level was associated with polymyxin B resistance, the survival rates of the strains challenged with polymyxin B were compared. The Δ*ugd *mutant producing the lowest amount of CPS was extremely sensitive to the treatment of polymyxin B (Figure 1C). Although the Δ*ugd *mutant was CPS-deficient, the impaired polymyxin resistance may have been largely attributed to the defect in LPS biosynthesis since the survival rates of Δ*wza *and Δ*rcsB *mutants appeared to be comparable with the parental strain CG43S3. This argues against the notion that the level of polymyxin B resistance is positively correlated to the amount of CPS [10]. Nevertheless, the possibility that a higher amount of CPS was required for the resistance could not be ruled out. As shown in Figure 1D, the introduction of pRK415-RcsB [39] resulted in a significantly higher resistance to polymyxin B in both Δ*rcsB *mutant and its parental strain. This indicated a protective effect of large amounts of CPS in polymyxin resistance.

### PmrF is involved in polymyxin B resistance and survival within macrophage

To investigate if the *K. pneumoniae pmr *homologues played a role in polymyxin B resistance, a *pmrF *deletion mutant strain and a plasmid pRK415-PmrF were generated. As shown in Figure 2A, when the strains were grown in LB medium, a low magnesium condition [40], differences in the survival rates were not apparent. When the strains were grown in LB supplemented with 1 mM FeCl_3_, an apparent deleting effect of *pmrF *in polymyxin B resistance was observed, and the survival rate could be restored by the introduction of pRK415-PmrF. The results indicated a role of PmrF in the polymyxin B resistance in high iron condition.

In addition to the mucosa surfaces, antimicrobial peptides and proteins play important roles in the microbicidal activity of phagosome [41]. To investigate the effect of *pmrF *deletion in the bacterial survival within phagosome, phagocytosis assay was carried out. Since *K. pneumoniae *CG43S3 was highly resistant to engulfment by phagocytes in our initial experiments, the Δ*rcsB *mutant which produced less CPS was used as the parental strain to generate Δ*pmrF*Δ*rcsB *mutant. As shown in Figure 2B, deletion of *pmrF *resulted in an approximately four-fold reduction in the recovery rate, which was restored after the introduction of pRK415-PmrF. This indicated an important role of *pmrF *not only in polymyxin B resistance but also in bacterial survival within macrophage.

### Deletion effect of *pmrA*, *pmrD *or *phoP *on polymyxin B resistance in *K. pneumoniae*

To investigate how PmrA, PhoP and PmrD were involved in the regulation of polymyxin B resistance in *K. pneumoniae*, Δ*pmrA*, Δ*phoP *and Δ*pmrD *mutant strains were generated. Deletion of either one of these genes resulted in a dramatic reduction of resistance to polymyxin B when the strains were grown in LB medium (Figure 3A). The deleting effects were no longer observed when the strains grown in LB supplemented with 10 mM magnesium, implying an involvement of the PhoP-dependent regulation in LB, a low magnesium environment. Under high-iron conditions, the deletion of *pmrA *caused the greatest reduction in the survival rate. Introduction of pRK415-PmrA or pRK415-PhoP into the Δ*pmrA*Δ*phoP *double mutant strain not only restored but also enhanced the bacterial resistance to polymyxin B (Figure 3B), which is likely due to an over-expression level of *phoP *or *pmrA *by the multicopy plasmid. Finally, whether the deletion of *pmrA*, *phoP *or *pmrD *affected the survival rate in phagosomes was also investigated. Interestingly, deletion of *phoP *resulted in most apparent effect while the *pmrA *deletion had less effect on the bacterial survival in macrophages. This was probably due to low iron concentration in the phagosomes [40]. The introduction of pRK415-PhoP or pRK415-PmrD could restore the recovery rates of Δ*phoP*Δ*rcsB *and Δ*pmrD*Δ*rcsB*, although not to the extent displayed by the parental strain. Taken together, our results indicate the presence of two independent pathways in the regulation of polymyxin B resistance and the bacterial survival within macrophage phagosomes.

### Effect of *pmrA*, *phoP *or *pmrD *deletion on P_*pmrH*_::*lacZ *or P_*pmrD*_::*lacZ *activity

**Figure 4:**
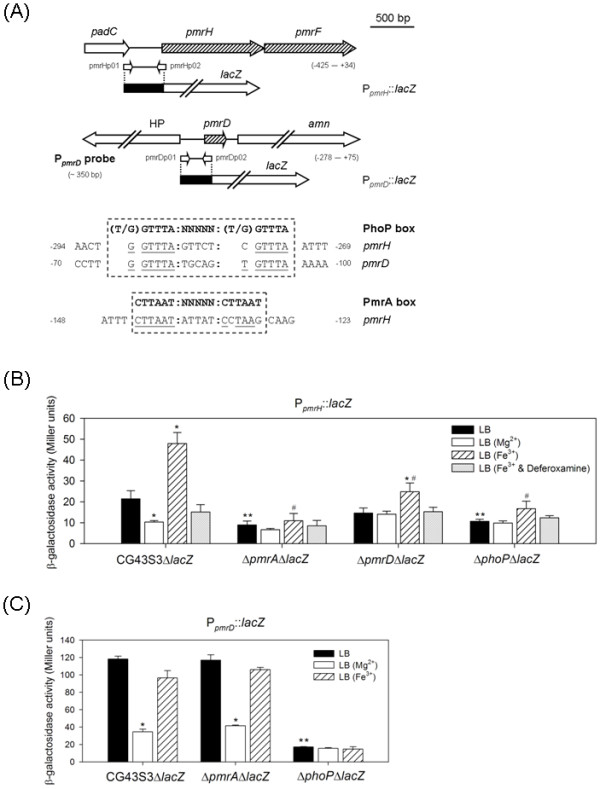
**Schematic representation of *pmrH *and *pmrD *loci and determination of *K. pneumoniae *P_*pmrH*_::*lacZ *and P_*pmrD*_::*lacZ *activity**. **(A) **Diagrammatic representation of the *pmrH *and *pmrD *loci. The large arrows represent the open reading frames. The relative positions of the primer sets used in PCR-amplification of the DNA fragments encompassing the P_*pmrH *_and P_*pmrD *_regions are indicated, and the numbers denote the relative positions to the translational start site. The name and approximate size of the DNA probes used in electro-mobility shift assay (EMSA) are shown on the left. The dashed boxes indicate the predicted PhoP and PmrA binding sequences and the alignment result is shown below. The identical nucleotide sequences are underlined. HP, hypothetical protein. **(B) **The β-galactosidase activities of log-phased cultures of *K. pneumoniae *strains carrying placZ15-PpmrH grown in LB, LB containing 10 mM MgCl_2_, LB containing 0.1 mM FeCl_3 _or 0.1 mM FeCl_3 _plusing 0.3 mM deferoxamine were determined and expressed as Miller units. **(C) **The β-galactosidase activities of log-phased cultures of *K. pneumoniae *strains carrying placZ15-PpmrD grown in LB, LB containing 10 mM MgCl_2 _or LB containing 0.1 mM FeCl_3 _were determined and expressed as Miller units. The data shown were the average ± standard deviations from triplicate samples. *, *P *< 0.01 compared to the same strain grown in LB medium. **, *P *< 0.01 compared to the parental strain grown in LB medium. #, *P *< 0.01 compared to the parental strain grown in LB medium supplemented with ferric ions.

As the functional role of the structural gene *pmrF *and the regulator genes *phoP*, *pmrD *and *pmrA *was verified, it would be of importance to investigate the regulatory network govern by PhoPQ-PmrD-PmrAB on the expression of *pmr *genes. Sequence analysis has revealed PhoP and PmrA box consensus in the upstream region of *pmrH *and PhoP box consensus in the upstream region of *pmrD *(Figure 4A). To investigate the interplay of PhoP, PmrA, and PmrD on the expression of *pmr *and *pmrD *genes, the reporter plasmids placZ15-PpmrH and placZ15-PpmrD were constructed and mobilized into *K. pneumoniae *CG43S3Δ*lacZ *and its derived Δ*pmrA*Δ*lacZ*, Δ*pmrD*Δ*lacZ *or Δ*phoP*Δ*lacZ *isogenic strains, respectively. The β-galactosidase activities of *K. pneumoniae *transformants under different environmental conditions were determined. In the wild-type strain CG43S3Δ*lacZ*, the P_*pmrH*_::*lacZ *activity was repressed in the presence of high magnesium but enhanced in high ferric ion (Figure 4B). Such iron-inducible activity was abolished after the addition of iron scavenger deferoxamine. As shown in Figure 4B, deleting effect of *pmrA *or *phoP *on the activity of P_*pmrH*_::*lacZ *could be observed in LB or LB supplemented with ferric iron. The negative effect of *pmrD *deletion was also apparent at high iron condition but was abolished after the addition of deferoxamine. The results clearly demonstrate the involvement of PmrA, PhoP and PmrD in the regulation of the expression of *pmr *genes, particularly in the presence of high ferric irons. As shown in Figure 4C, the P_*pmrD*_::*lacZ *activity was significantly reduced in high-magnesium conditions or upon the deletion of *phoP*. Interestingly, the deletion of *pmrA *or high ferric irons had little effect on the activity of P_*pmrD*_::*lacZ*. The results suggest that the expression of *K. pneumoniae pmrD *is regulated in a PhoP-dependent but PmrA-independent manner.

### Analysis of EMSA indicates a direct binding of the recombinant PhoP to _*pmrD*_

**Figure 5:**
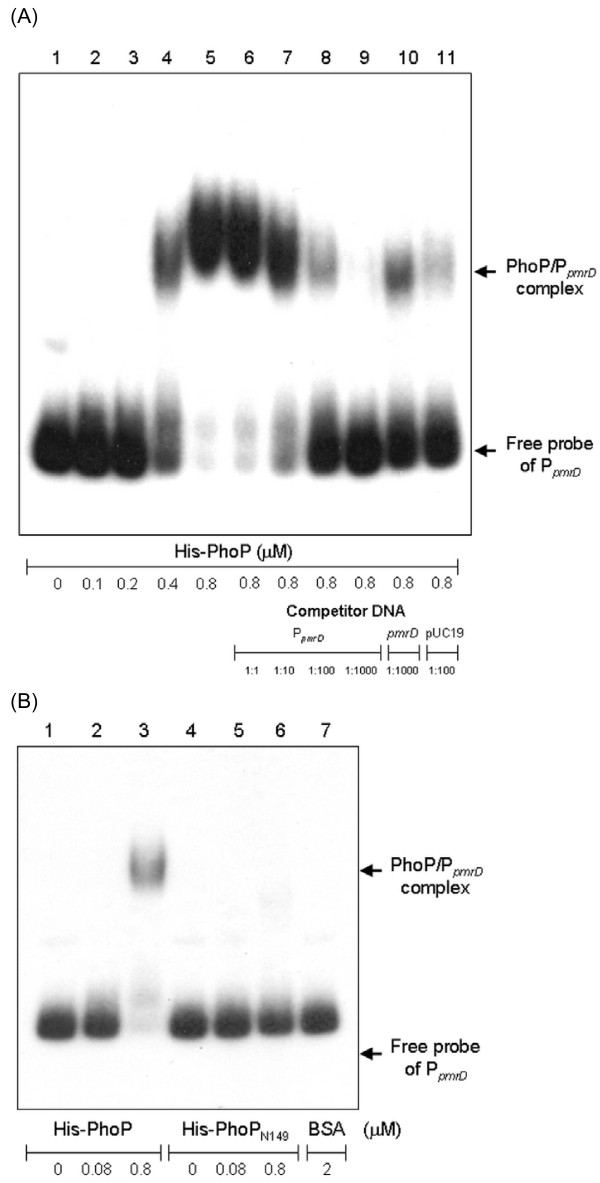
**Binding of His-PhoP and His-PhoP_N149 _to P_*pmrD*_**. **(A) **Specific binding of recombinant His-PhoP protein to the putative *pmrD *promoter. EMSA was performed by using the ^32^P-labeled DNA probe of P_*pmrD *_incubated with increasing amounts of the His-PhoP (lanes 2 to 5), with 40 pmole of His-PhoP plus increasing amounts of the unlabeled P_*pmrD *_DNA (specific competitor, lane 6 to 9), or with excess amounts of non-specific competitor DNA (lane 10 and 11). The amounts of recombinant proteins and DNA probes used are indicated in the figure. **(B) **EMSA was performed with 0, 4 or 40 pmole of His-PhoP (lanes 1 to 3), His-PhoP_N149 _(lanes 4 to 6) or 100 pmole of BSA (lane 7). The arrows indicate the PhoP/P_*pmrD *_complex and free probe of P_*pmrD*_.

The binding of PhoP or PmrA to P_*pmrH *_has been determined recently [31]. In order to determine whether PhoP binds directly to P_*pmrD*_, EMSA was performed. As shown in Figure 5A, binding of the recombinant His-PhoP protein to P_*pmrD *_was evident by the formation of a protein/DNA complex with a slower mobility. The binding specificity was also examined by the addition of specific DNA competitor or non-specific DNA competitor. As shown in Figure 5B, the formation of protein/DNA complex diminished when His-PhoP_N149_, in which the carboxyl-terminal helix-turn-helix domain has been truncated, was used instead of His-PhoP. The results strongly suggest the PhoP binds via its C-terminal domain to the promoter of *pmrD *for the activation of the *pmrD *expression in *K. pneumoniae*.

### Two-hybrid analysis of the *in vivo *interaction between *Klebsiella *PmrD and PmrA

**Figure 6:**
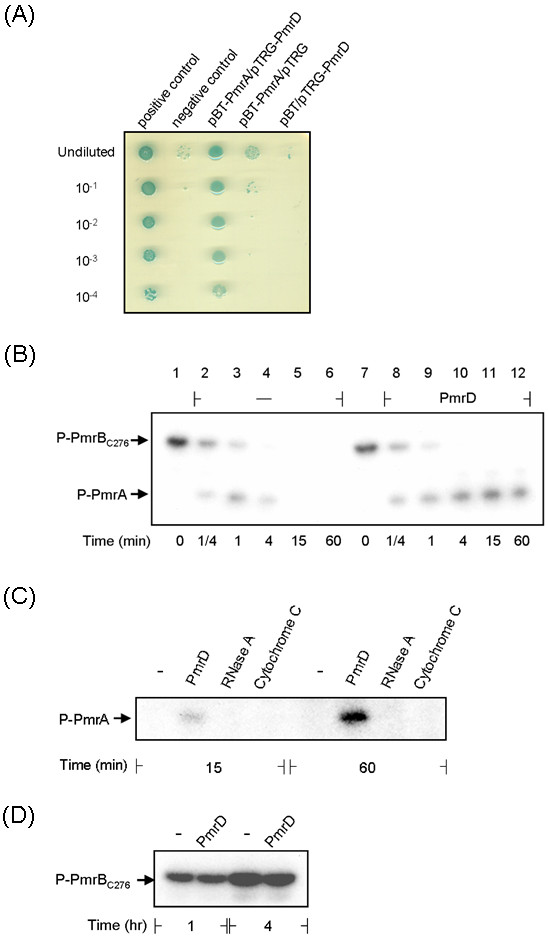
***Klebsiella *PmrD interacts with PmrA to prevent dephosphorylation**. **(A) **Bacterial two-hybrid analysis of PmrD/PmrA interaction *in vivo*. The *E. coli *XL1-Blue cells co-transformed with various combinations of pTRG and pBT-derived plasmids were diluted serially and spotted onto the indicator plate. The bacterial growth after 36 h was investigated and photographed. Combinations of plasmids carried by each strain were indicated above the figure. **(B) ***Klebsiella *PmrD prevents the dephosphorylation of PmrA by its cognate sensor protein. The phosphorylation state of the recombinant His-PmrA protein was monitored upon the addition of the sensor protein His-PmrB_C276 _in the presence (PmrD) or absence (-) of purified His-PmrD protein at specific time points as indicated. The arrows indicate phospho-PmrA (P-PmrA) and phospho-PmrB_C276 _(P-PmrB_C276_). **(C) **Kinase/phosphatase assay was carried out using the recombinant His-PmrA (final concentration 5 μM) and His-PmrB_C276 _(final concentration 2.5 μM) in the presence (PmrD) or absence (-) of the recombintant His-PmrD protein (final concentration 5 μM). The small cationic proteins RNase A and cytochrome C were introduced individually as a negative control at a final concentration of 5 μM. **(D) **Autokinase assay of the recombinant His-PmrB_C276 _(final concentration 2.5 μM) was performed in the presence (PmrD) or absence (-) of the recombintant His-PmrD protein (final concentration 5 μM).

The interaction between *Klebsiella *PmrD and PmrA has been shown as a prerequisite for the connector-mediated pathway [31]. To demonstrate *in vivo *interaction, a bacterial two-hybrid assay was performed. The plasmid pBT-PmrA carrying the RNAPα-PmrA coding region and the plasmid pTRG-PmrD carrying the λ-cI-PmrD coding sequence were generated. *In vivo *interaction between the two reporter strains allowed the binding of λ-cI to the operator region as well as the recruitment of α-RNAP for the expression of the *ampR *and *lacZ *reporter genes. The bacteria harboring the positive control plasmids pTRG-Gal11^P^/pBT-LGF2 showed a more vigorous growth on the indicator plate, as reflected by the apparent colony formation when the culture was diluted serially (Figure 6A). In contrast, the strain carrying the negative control vectors pBT/pTRG revealed impaired colony formation. As shown in Figure 6A, a similar growth pattern of the *E. coli *cells harboring pBT-PmrA/pTRG-PmrD to that of the positive control cells was observed indicating an interaction between the PmrD and PmrA.

### The PmrD binds to PmrA to prevent dephosphorylation

In *S. enterica*, the phosphorylation of PmrA by the cognate sensor protein PmrB has been demonstrated to enhance its affinity in binding to its target promoter. The subsequent dephosphorylation of PmrA by PmrB helped to relieve from over-activation of this system (1). In *Salmonella*, PmrD has been shown to be able to protect PmrA from both intrinsic and PmrB-mediated dephosphorylation (22). To verify if *Klebsiella *PmrD also participates in the phosphorylation, *in vitro *phosphotransfer assay was carried out with the recombinant proteins His-PmrA, His-PmrD and His-PmrB_C276_. As shown in Figure 6B, the His-PmrA was rapidly phosphorylated upon addition of the autophosphorylated His-PmrB_C276 _and then gradually dephosphorylated. Addition of His-PmrD apparently prolong the phosphorylation state of the His-PmrA, which could be maintained for at least 60 min (Figure 6B). The phosphorylated His-PmrA appeared to be very stable in the presence of the His-PmrD since the phosphorylation signal was still detectable 4 h later (data not shown). As shown in Figure 6C, the specificity of the interaction between His-PmrD and His-PmrA was also demonstrated since the phosphorylation state of His-PmrA could not be detected when incubated with the small cationic proteins RNase A or cytochrome C [30]. Similar levels of phospho-PmrB_C276 _were observed in the presence or absence of PmrD (Figure 6D), suggesting the His-PmrD had no effect on the phorphorylation state of His-PmrB_C276_.

## Discussion

Although the amount of CPS produced by Δ*rcsB *mutant was more than twice of that produced by Δ*wza *mutant, no apparent difference between the wild type strain CG43S3, Δ*wza *mutant, and Δ*rcsB *mutant in polymyxin B resistance could be observed. This is different from the previous finding that *K. pneumoniae *CPS was an important physical barrier for the APs [10]. This discrepancy may be attributed to some of the *K. pneumoniae *strains used for comparison in the previous study produced extremely low level of the CPS. Nevertheless, a higher amount of CPS was protective for the bacterial resistance to polymyxin B.

On the other hand, the deletion of *ugd *resulted in the loss of resistance to polymyxin B. Sequence analysis of the available *K. pneumoniae *genome NTHU-K2044 [42], MGH78578 (http://genome.wustl.edu/) and 342 [43] revealed no PmrA [17] or PhoP box [27] in the upstream region of the *manC*-*manB*-*ugd *genes [44]. This implies the involvement of a regulatory mechanism different from that for *S. enterica ugd*, which was positively regulated by the three 2CS regulators PhoP, PmrA and RcsB [45].

Consistent with the reported findings [31], deletion of *Klebsiella pmrF *which encodes one of the enzymes required for synthesis and incorporation of aminoarabinose in LPS resulted in decreased resistance to polymyxin B and survival within macrophages. The *pmr *expression has been shown to be directly regulated by PhoP under low magnesium or by PmrA in high ferric ions, or by the connector-mediated pathway reported for *Salmonella*,[31]. Similar to the observations in *E. coli*, *S. enterica *[25], *Yersinia pestis *[46], and *Pseudomonas aeruginosa *[47], a positive regulatory role of PmrA and PhoP in polymyxin B resistance in *K. pneumoniae *was also demonstrated.

The deletion of *phoP *resulted in more drastic effect on the bacterial survival in macrophage than the *pmrA *deletion, implying a different level of control between PhoP and PmrA in *K. pneumoniae *resistance to phagocytosis. During phagocytosis, phagosomal maturation and phagolysosomes formation are accompanied by progressive acidification and acquisition of various hydrolases, reactive oxygen, nitrogen species, and APs [48]. Low pH and low-magnesium have been shown to be able to stimulate expression of the PhoP-activated genes [40,49]. Apart from its microbicidal activity, the APs inside phagosomes has even been reported as an inducing signal for the activation of the PhoP/PhoQ system [50]. The deletion of *pmrF *or *phoP *caused a significant reduction in intramacrophage survival of the bacterial, implying a role of the AP resistance regulation in the bacterial pathogenesis.

Until now, PmrD was only found in *E. coli*, *Shigella flexneri*, *S. enterica *and *K. pneumoniae*. Although PmrD in *Klebsiella *appeared to act in a way similar to the PmrD in *S. enterica*, they share only about 40% sequence identity. The expression of *K. pneumoniae pmrD *was shown to be PhoP-dependent and the regulation was achieved through a direct binding of PhoP to the putative *pmrD *promoter. In addition, the binding of PmrD was shown to efficiently protect the PmrA from dephosphorylation. The *in vivo *interaction between PmrD and PmrA demonstrated using 2-hybrid analysis further supported the presence of the connector-mediated pathway in *K. pneumoniae*.

In summary, involvement of *Klebsiella pmr *in polymyxin B resistance and the regulation for the expression of *pmr *genes were analyzed. The regulatory network for the expression of the *pmr *genes is comprised of 2CS response regulators PhoP and PmrA, and the connector protein PmrD. The demonstration of PmrD in prolonging the phosphorylation state of phosphor-PmrA further confirmed the presence of a connector-mediated pathway in *K. pneumoniae*. The complexity in the control of *pmr *genes expression may provide ecological niches for *K. pneumoniae *in response to a variety of environmental clues; for example, in the process of infection.

## Competing interests

The authors declare that they have no competing interests.

## Authors' contributions

HYC conceived the study, designed and performed the experiments, interpreted the data, drafted and revised the manuscript. YFC helped with the polymyxin B resistance assay. HLP coordinated the study and revised the manuscript for scientific content as the corresponding author. All authors have read and approved the final manuscript.

## References

[B1] Podschun R Ullmann U Klebsiella spp. as nosocomial pathogens: epidemiology, taxonomy, typing methods, and pathogenicity factors Clin Microbiol Rev 1998 11 589 603 9767057 10.1128/cmr.11.4.589 PMC88898

[B2] Falagas ME Kopterides P Old antibiotics for infections in critically ill patients Current opinion in critical care 2007 13 592 597 10.1097/MCC.0b013e32827851d7 17762241

[B3] Falagas ME Bliziotis IA Pandrug-resistant Gram-negative bacteria: the dawn of the post-antibiotic era? Int J Antimicrob Agents 2007 29 630 636 10.1016/j.ijantimicag.2006.12.012 17306965

[B4] Kasiakou SK Michalopoulos A Soteriades ES Samonis G Sermaides GJ Falagas ME Combination therapy with intravenous colistin for management of infections due to multidrug-resistant Gram-negative bacteria in patients without cystic fibrosis Antimicrob Agents Chemother 2005 49 3136 3146 10.1128/AAC.49.8.3136-3146.2005 16048915 PMC1196256

[B5] Zavascki AP Goldani LZ Li J Nation RL Polymyxin B for the treatment of multidrug-resistant pathogens: a critical review The Journal of antimicrobial chemotherapy 2007 60 1206 1215 10.1093/jac/dkm357 17878146

[B6] Hancock RE Peptide antibiotics Lancet 1997 349 418 422 10.1016/S0140-6736(97)80051-7 9033483

[B7] Kabha K Nissimov L Athamna A Keisari Y Parolis H Parolis LA Grue RM Schlepper-Schafer J Ezekowitz AR Ohman DE Relationships among capsular structure, phagocytosis, and mouse virulence in Klebsiella pneumoniae Infect Immun 1995 63 847 852 7868255 10.1128/iai.63.3.847-852.1995 PMC173080

[B8] Domenico P Salo RJ Cross AS Cunha BA Polysaccharide capsule-mediated resistance to opsonophagocytosis in Klebsiella pneumoniae Infect Immun 1994 62 4495 4499 7927714 10.1128/iai.62.10.4495-4499.1994 PMC303135

[B9] Cortes G Borrell N de Astorza B Gomez C Sauleda J Alberti S Molecular analysis of the contribution of the capsular polysaccharide and the lipopolysaccharide O side chain to the virulence of Klebsiella pneumoniae in a murine model of pneumonia Infect Immun 2002 70 2583 2590 10.1128/IAI.70.5.2583-2590.2002 11953399 PMC127904

[B10] Campos MA Vargas MA Regueiro V Llompart CM Alberti S Bengoechea JA Capsule polysaccharide mediates bacterial resistance to antimicrobial peptides Infect Immun 2004 72 7107 7114 10.1128/IAI.72.12.7107-7114.2004 15557634 PMC529140

[B11] Whitfield C Paiment A Biosynthesis and assembly of Group 1 capsular polysaccharides in Escherichia coli and related extracellular polysaccharides in other bacteria Carbohydr Res 2003 338 2491 2502 10.1016/j.carres.2003.08.010 14670711

[B12] Drummelsmith J Whitfield C Translocation of group 1 capsular polysaccharide to the surface of Escherichia coli requires a multimeric complex in the outer membrane EMBO J 2000 19 57 66 10.1093/emboj/19.1.57 10619844 PMC1171777

[B13] Majdalani N Gottesman S The Rcs phosphorelay: a complex signal transduction system Annu Rev Microbiol 2005 59 379 405 10.1146/annurev.micro.59.050405.101230 16153174

[B14] Lai YC Peng HL Chang HY RmpA2, an activator of capsule biosynthesis in Klebsiella pneumoniae CG43, regulates K2 cps gene expression at the transcriptional level J Bacteriol 2003 185 788 800 10.1128/JB.185.3.788-800.2003 12533454 PMC142793

[B15] Kim SH Jia W Parreira VR Bishop RE Gyles CL Phosphoethanolamine substitution in the lipid A of Escherichia coli O157: H7 and its association with PmrC Microbiology (Reading, England) 2006 152 657 666 16514146 10.1099/mic.0.28692-0

[B16] Gunn JS Ryan SS Van Velkinburgh JC Ernst RK Miller SI Genetic and functional analysis of a PmrA-PmrB-regulated locus necessary for lipopolysaccharide modification, antimicrobial peptide resistance, and oral virulence of Salmonella enterica serovar typhimurium Infect Immun 2000 68 6139 6146 10.1128/IAI.68.11.6139-6146.2000 11035717 PMC97691

[B17] Wosten MM Groisman EA Molecular characterization of the PmrA regulon The Journal of biological chemistry 1999 274 27185 27190 10.1074/jbc.274.38.27185 10480935

[B18] Yan A Guan Z Raetz CR An undecaprenyl phosphate-aminoarabinose flippase required for polymyxin resistance in Escherichia coli The Journal of biological chemistry 2007 282 36077 36089 10.1074/jbc.M706172200 17928292 PMC2613183

[B19] Breazeale SD Ribeiro AA McClerren AL Raetz CR A formyltransferase required for polymyxin resistance in Escherichia coli and the modification of lipid A with 4-Amino-4-deoxy-L-arabinose. Identification and function oF UDP-4-deoxy-4-formamido-L-arabinose The Journal of biological chemistry 2005 280 14154 14167 10.1074/jbc.M414265200 15695810

[B20] Marceau M Sebbane F Ewann F Collyn F Lindner B Campos MA Bengoechea JA Simonet M The pmrF polymyxin-resistance operon of Yersinia pseudotuberculosis is upregulated by the PhoP-PhoQ two-component system but not by PmrA-PmrB, and is not required for virulence Microbiology (Reading, England) 2004 150 3947 3957 15583148 10.1099/mic.0.27426-0

[B21] Tamayo R Ryan SS McCoy AJ Gunn JS Identification and genetic characterization of PmrA-regulated genes and genes involved in polymyxin B resistance in Salmonella enterica serovar typhimurium Infect Immun 2002 70 6770 6778 10.1128/IAI.70.12.6770-6778.2002 12438352 PMC133008

[B22] Lacour S Bechet E Cozzone AJ Mijakovic I Grangeasse C Tyrosine phosphorylation of the UDP-glucose dehydrogenase of Escherichia coli is at the crossroads of colanic acid synthesis and polymyxin resistance PLoS One 2008 3 e3053 10.1371/journal.pone.0003053 18725960 PMC2516531

[B23] Wosten MM Kox LF Chamnongpol S Soncini FC Groisman EA A signal transduction system that responds to extracellular iron Cell 2000 103 113 125 10.1016/S0092-8674(00)00092-1 11051552

[B24] Gunn JS The Salmonella PmrAB regulon: lipopolysaccharide modifications, antimicrobial peptide resistance and more Trends Microbiol 2008 16 284 290 10.1016/j.tim.2008.03.007 18467098

[B25] Winfield MD Groisman EA Phenotypic differences between Salmonella and Escherichia coli resulting from the disparate regulation of homologous genes Proc Natl Acad Sci USA 2004 101 17162 17167 10.1073/pnas.0406038101 15569938 PMC534605

[B26] Perez JC Groisman EA Acid pH activation of the PmrA/PmrB two-component regulatory system of Salmonella enterica Molecular microbiology 2007 63 283 293 10.1111/j.1365-2958.2006.05512.x 17229213 PMC1804205

[B27] Kato A Tanabe H Utsumi R Molecular characterization of the PhoP-PhoQ two-component system in Escherichia coli K-12: identification of extracellular Mg2+-responsive promoters J Bacteriol 1999 181 5516 5520 10464230 10.1128/jb.181.17.5516-5520.1999 PMC94065

[B28] Kox LF Wosten MM Groisman EA A small protein that mediates the activation of a two-component system by another two-component system Embo J 2000 19 1861 1872 10.1093/emboj/19.8.1861 10775270 PMC302009

[B29] Kato A Latifi T Groisman EA Closing the loop: the PmrA/PmrB two-component system negatively controls expression of its posttranscriptional activator PmrD Proc Natl Acad Sci USA 2003 100 4706 4711 10.1073/pnas.0836837100 12676988 PMC153620

[B30] Kato A Groisman EA Connecting two-component regulatory systems by a protein that protects a response regulator from dephosphorylation by its cognate sensor Genes Dev 2004 18 2302 2313 10.1101/gad.1230804 15371344 PMC517523

[B31] Mitrophanov AY Jewett MW Hadley TJ Groisman EA Evolution and dynamics of regulatory architectures controlling polymyxin B resistance in enteric bacteria PLoS genetics 2008 4 e1000233 10.1371/journal.pgen.1000233 18949034 PMC2565834

[B32] Chang HY Lee JH Deng WL Fu TF Peng HL Virulence and outer membrane properties of a galU mutant of Klebsiella pneumoniae CG43 Microb Pathog 1996 20 255 261 10.1006/mpat.1996.0024 8861391

[B33] Peng HL Wang PY Wu JL Chiu CT Chang HY Molecular epidemiology of Klebsiella pneumoniae Zhonghua Min Guo Wei Sheng Wu Ji Mian Yi Xue Za Zhi 1991 24 264 271 1726368

[B34] Skorupski K Taylor RK Positive selection vectors for allelic exchange Gene 1996 169 47 52 10.1016/0378-1119(95)00793-8 8635748

[B35] Lin CT Huang TY Liang WC Peng HL Homologous response regulators KvgA, KvhA and KvhR regulate the synthesis of capsular polysaccharide in Klebsiella pneumoniae CG43 in a coordinated manner J Biochem (Tokyo) 2006 140 429 438 10.1093/jb/mvj168 16877448

[B36] Keen NT Tamaki S Kobayashi D Trollinger D Improved broad-host-range plasmids for DNA cloning in gram-negative bacteria Gene 1988 70 191 197 10.1016/0378-1119(88)90117-5 2853689

[B37] Domenico P Schwartz S Cunha BA Reduction of capsular polysaccharide production in Klebsiella pneumoniae by sodium salicylate Infect Immun 1989 57 3778 3782 2680983 10.1128/iai.57.12.3778-3782.1989 PMC259904

[B38] Miller JH Experiments in Molecular Genetics 1972 Cold Spring Harbor Laboratory Press, Cold Spring Harbor, N.Y.

[B39] Cheng HY Chen YS Wu CY Chang HY Lai YC Peng HL RmpA regulation of capsular polysaccharide biosynthesis in Klebsiella pneumoniae CG43 J Bacteriol 192 3144 3158 10.1128/JB.00031-10 20382770 PMC2901686

[B40] Groisman EA The ins and outs of virulence gene expression: Mg2+ as a regulatory signal Bioessays 1998 20 96 101 10.1002/(SICI)1521-1878(199801)20:1<96::AID-BIES13>3.0.CO;2-3 9504051

[B41] Benincasa M Mattiuzzo M Herasimenka Y Cescutti P Rizzo R Gennaro R Activity of antimicrobial peptides in the presence of polysaccharides produced by pulmonary pathogens J Pept Sci 2009 15 595 600 10.1002/psc.1142 19466693

[B42] Wu KM Li LH Yan JJ Tsao N Liao TL Tsai HC Fung CP Chen HJ Liu YM Wang JT Fang CT Chang SC Shu HY Liu TT Chen YT Shiau YR Lauderdale TL Su IJ Kirby R Tsai SF Genome Sequencing and Comparative Analysis of Klebsiella pneumoniae NTUH-K2044, a Strain Causing Liver Abscess and Meningitis Journal of bacteriology 2009 19447910 10.1128/JB.00315-09 PMC2704730

[B43] Fouts DE Tyler HL DeBoy RT Daugherty S Ren Q Badger JH Durkin AS Huot H Shrivastava S Kothari S Dodson RJ Mohamoud Y Khouri H Roesch LF Krogfelt KA Struve C Triplett EW Methé BA Complete genome sequence of the N2-fixing broad host range endophyte Klebsiella pneumoniae 342 and virulence predictions verified in mice PLoS genetics 2008 4 e1000141 10.1371/journal.pgen.1000141 18654632 PMC2453333

[B44] Chuang YP Fang CT Lai SY Chang SC Wang JT Genetic determinants of capsular serotype K1 of Klebsiella pneumoniae causing primary pyogenic liver abscess J Infect Dis 2006 193 645 654 10.1086/499968 16453259

[B45] Mouslim C Groisman EA Control of the Salmonella ugd gene by three two-component regulatory systems Molecular microbiology 2003 47 335 344 10.1046/j.1365-2958.2003.03318.x 12519186

[B46] Winfield MD Latifi T Groisman EA Transcriptional regulation of the 4-amino-4-deoxy-L-arabinose biosynthetic genes in Yersinia pestis J Biol Chem 2005 280 14765 14772 10.1074/jbc.M413900200 15710615

[B47] Moskowitz SM Ernst RK Miller SI PmrAB, a two-component regulatory system of Pseudomonas aeruginosa that modulates resistance to cationic antimicrobial peptides and addition of aminoarabinose to lipid A J Bacteriol 2004 186 575 579 10.1128/JB.186.2.575-579.2004 14702327 PMC305751

[B48] Flannagan RS Cosio G Grinstein S Antimicrobial mechanisms of phagocytes and bacterial evasion strategies Nat Rev Microbiol 2009 7 355 366 10.1038/nrmicro2128 19369951

[B49] Groisman EA The pleiotropic two-component regulatory system PhoP-PhoQ J Bacteriol 2001 183 1835 1842 10.1128/JB.183.6.1835-1842.2001 11222580 PMC95077

[B50] Otto M Bacterial sensing of antimicrobial peptides Contrib Microbiol 2009 16 136 149 full_text 19494583 10.1159/000219377 PMC2777530

